# Resting-state brain networks alterations in adolescents with Internet Gaming Disorder associate with cognitive control impairments

**DOI:** 10.3389/fpsyt.2024.1404050

**Published:** 2024-09-09

**Authors:** Tao Zhao, Yibo Zhang, Yange Li, Jie Wu, Ruiqi Wang, Qiyan Lv, Dingyi Li, Yan Lang

**Affiliations:** Department of Psychiatry, First Affiliated Hospital of Zhengzhou University, Zhengzhou, Henan, China

**Keywords:** Internet Gaming Disorder, adolescents, independent component analysis, resting-state brain networks, cognitive control

## Abstract

**Objective:**

Research indicates that cognitive control is compromised in individuals with internet gaming disorder (IGD). However, the neural mechanisms behind it are still unclear. This study aims to investigate alterations in resting-state brain networks in adolescents with IGD and the potential neurobiological mechanisms underlying cognitive dysfunction.

**Materials and methods:**

A total of 44 adolescent IGD subjects (male/female: 38/6) and 50 healthy controls (male/female: 40/10) were enrolled. Participants underwent demographic assessments, Young’s Internet Addiction Scale, Barratt Impulsiveness Scale 11 Chinese Revised Version, the Chinese Adolescents’ Maladaptive Cognitions Scale, exploratory eye movement tests, and functional magnetic resonance imaging (fMRI). FMRI data were analyzed using the GIFT software for independent component analysis, focusing on functional connectivity within and between resting-state brain networks.

**Results:**

In comparison to the control group, impulsivity in adolescent IGD subjects showed a positive correlation with the severity of IGD (r=0.6350, p < 0.001), linked to impairments in the Executive Control Network (ECN) and a decrease in functional connectivity between the Salience Network (SN) and ECN (r=0.4307, p=0.0021; r=-0.5147, p=0.0034). Decreased resting state activity of the dorsal attention network (DAN) was associated with attentional dysregulation of IGD in adolescents (r=0.4071, p=0.0017), and ECN increased functional connectivity with DAN. The degree of IGD was positively correlated with enhanced functional connectivity between the ECN and DAN (r=0.4283, p=0.0037).

**Conclusions:**

This research demonstrates that changes in the ECN and DAN correlate with heightened impulsivity and attentional deficits in adolescents with IGD. The interaction between cognitive control disorders and resting-state brain networks in adolescent IGD is related.

## 1 Introduction

Internet Gaming Disorder (IGD) is characterized as a distinct behavioral addiction marked by the excessive and compulsive engagement with video games (1). Recent epidemiological studies (2, 3) have shown that the morbidity of IGD among adolescents is approximately 5.5%. In China’s urban regions, it is estimated that approximately 14% of adolescents are affected by IGD, totaling around 24 million individuals (3, 4). The notion of Internet Gaming Disorder (IGD) was initially introduced in the fifth edition of the Diagnostic and Statistical Manual of Mental Disorders (DSM-5) (5). In 2018, the 11th revision of the International Classification of Diseases (ICD-11) officially recognized gaming disorder as a mental disorder stemming from addictive behaviors (6). Cognitive control deficits are viewed as a significant risk factor for behavioral addictions, characterized by inadequate emotional regulation, compromised cognitive skills, attention deficits, and impulsive behavior. The progression of IGD further deteriorates an individual’ s cognitive capacities, creating a vicious cycle (7, 8). Currently, there is a lack of high-quality evidence-supported effective treatment measures (9), which may be related to the still unknown pathophysiology and cognitive mechanisms of IGD (10).

A neural network model derived from functional magnetic resonance imaging (fMRI) data holds promise for elucidating the pathogenesis of IGD. Menon et al. proposed the triple network model (11). The salience network (SN) is believed to be associated with detection and coordination (12), including regulating the activities of the default mode network (DMN) and the executive control network (ECN). DMN is active during an individual’s resting state, participating in processes such as introspection, memory integration, emotional processing, and social cognition (13). ECN participating in multiple advanced cognitive tasks and playing an important role in cognitive control. Networks are widely connected and influence each other. The neurobiological mechanisms of various mental disorders, including addiction, can be explained using a triple network model (14). However, there is less research on other intrinsic connectivity networks (ICNs) (15), although these ICNs have been proven to be related to cognitive functions (16), such as the dorsal attention network (DAN) involved in top-down action and perception processes and attention control, and the ventral attention network (VAN) related to stimulus-driven attention control. In network analysis techniques, the independent component analysis (ICA) method allows for better identification of ICNs, and then studying how functional connectivity related to these networks is regulated (17).

Zhang et al. found that compared with HCs, IGD had significantly increased SN-DMN connectivity, suggest that the deficient modulation of ECN versus DMN by SN (18). One study suggested that the diminished cognitive control during real-time gameplay was associated with FC alterations, involving a weak FC in the cognitive control network, suggesting that individuals with IGD may have less cognitive control (19).Recent resting-state fMRI studies indicating that IGDs show enhanced rsFC between the ventral attention network and regions within the somatomotor network, suggesting that the interaction between stimuli-driven attention and addictive behavior might be facilitated (20, 21). However, The specific pathological mechanisms of these network interactions in IGD cognitive control disorders are uncertain and therefore require more research. Since the prevalence of IGD is highest during adolescence (2), the developmental characteristics of the brain in adolescents should be considered when exploring the pathogenesis of IGD. The Executive Control Network is still not fully developed during adolescence (22), so it may play a different role in the neural mechanisms of adolescent IGD compared to adult IGD. Diverging from prior research, this study investigates alterations within and among resting-state brain networks, emphasizing the association between these changes in brain networks and impaired cognitive control abilities. It is the first study to examine the role of modifications in the ECN in the mechanism of IGD in adolescents.

In this study, we examined the resting-state functional connectivity within and between ICNs in adolescents with IGD and HC. We analyzed the differences in internal interactions of ICNs between the IGD and HC groups and assessed the functional connectivity between ICNs. Drawing on prior resting-state functional magnetic resonance imaging (rs-fMRI) research, we investigated the DMN, ECN, SN, VAN, and DAN. We hypothesized that compared to the HC group, the functional connectivity between these networks would be reduced in adolescent IGD, indicating neurodevelopmental changes that may be associated with cognitive dysfunction.

## 2 Materials and methods

### 2.1 Study participants

The study recruited adolescent participants from the outpatient and inpatient units of the Department of Psychiatry at the First Affiliated Hospital of Zhengzhou University between September 2021 and September 2023. Inclusion Criteria for the IGD Group: 1. Age range of 12-18 years, regardless of gender. 2. Asian ethnicity and right-handedness. 3. Diagnosis of internet gaming disorder as per DSM-5 criteria by a psychiatrist. 4. A total score of ≥40 on Young’s Internet Addiction Scale. Inclusion Criteria for the HC Group: 1. Age range of 12-18 years, regardless of gender. 2. Asian ethnicity and right-handedness. 3. A total score of <40 on Young’s Internet Addiction Scale. Common Exclusion Criteria: 1. History of severe brain trauma or organic brain diseases (e.g. encephalitis, epilepsy). 2. Mental retardation. 3. Comorbid or past history of psychiatric disorders (e.g. schizophrenia, depression, anxiety disorders, bipolar disorder). 4. Family history of psychiatric illnesses or hereditary diseases. 5. History of substance or drug abuse. 6. Contraindications to MRI scanning. Additional Exclusion Criteria for the IGD Group: 1. Use of psychiatric medications or other treatments such as physical therapy or psychotherapy within the past month. The study received approval from the Ethics Committee of the First Affiliated Hospital of Zhengzhou University and was conducted in accordance with the Declaration of Helsinki. Informed consent was obtained from all participants and/or their guardians, who signed the consent form.

### 2.2 Methodology

#### 2.2.1 General data collection

Psychiatrists evaluated all study subjects based on the IGD criteria to determine their eligibility and exclusion. Basic information, including gender, age, educational background, ethnicity, history of mental and physical health conditions, and family history, was gathered from all participants. Moreover, participants were assessed using various scales, and their rs-fMRI data were collected.

#### 2.2.2 Psychological scale assessments

Young’s Internet Addiction Scale: All subjects were evaluated using the Young’s Internet Addiction Scale (23), a 20-item questionnaire scored on a 5-point scale, resulting in a maximum score of 100. The scale categorizes addiction severity into mild (40 ≤ Young score < 60), moderate (60 ≤ Young score < 80), and severe (Young score ≥ 80), with higher scores indicating more severe internet gaming disorder.

Barratt Impulsiveness Scale 11 Chinese Revised Version (BIS-11): The BIS-11 (24) is composed of three subscales: cognitive impulsiveness, motor impulsiveness, and non-planning impulsiveness. Each subscale has scores ranging from 10 to 50, where higher scores denote increased impulsivity.

The Chinese Adolescents’ Maladaptive Cognitions Scale (CAMCS) (25): This scale comprises 12 items, each rated on a Likert scale from 1 (strongly agree) to 5 (strongly disagree), with higher scores reflecting greater maladaptive cognitions. It demonstrates good internal consistency (alpha = 0.81) and validity.

The scales were filled out by the patient themselves, and the evaluation work was completed by a professional psychiatrist from the First Affiliated Hospital of Zhengzhou University.

#### 2.2.3 Exploratory eye movements monitoring

All subjects participated in EEM testing utilizing the Shanghai Dekang DEM-2000 eye movement detection system. EEM studies may facilitate understanding of the neurobiology of populations with mental disorders, and evaluate mechanisms involved in attention processes. For example, reflexive saccades are considered to be type of cognitive parameter that evaluates attention (26). In this study, assess the presence of attentional deficit in subjects by analyzing the number of eye fixations (NEF), search score responses (RSS), and discriminant (D) values within the initial 15 seconds of observing a target image.

#### 2.2.4 MRI data collection

MRI data were acquired using a Siemens Magnetom Prisma 3.0T MRI scanner equipped with a 64-channel head coil. Participants were advised to lie in a supine position, keep their eyes open, breathe calmly, and refrain from any spontaneous mental activities. Initially, a standard MRI head scan sequence was conducted to eliminate individuals with notable brain structural abnormalities. Subsequently, rs-fMRI scans were performed utilizing single-shot echo-planar imaging (EPI) technology. The detailed scanning sequences and parameters were as follows: 1. Routine MRI scan: T1-weighted imaging (T1WI) sequence with a repetition time (TR) of 190 ms, echo time (TE) of 2.6 ms, interslice spacing of 1 mm, slice thickness of 5 mm, flip angle of 70°, and field of view (FOV) of 240 mm × 240 mm, covering 20 slices. Rs-fMRI scans were performed on subjects with no abnormal T1WI sequence. 2. Rs-fMRI scan: Blood-oxygen-level-dependent (BOLD) sequence with a TR of 1000 ms, TE of 30 ms, flip angle of 70°, slice thickness of 2.2 mm, covering 52 slices. The total scan duration was 360 seconds.

#### 2.2.5 fMRI data preprocessing

The preprocessing of data was conducted using the DPABI toolkit on the MATLAB platform, involving the following steps: (1) Conversion of data format: Transforming files from DICOM to NIFTI format. (2) Elimination of initial images: Discarding the first 10 time points to mitigate noise in the early scan phase. (3) Timing of slices: Adjusting for differences in timing between slices, using the middle slice as the reference point. (4) Realignment: Excluding participants with head movement exceeding 3 mm in displacement or 3° in rotation; however, no participants were excluded for excessive head motion in this study. (5) Normalization: Adapting rs-fMRI images to fit the EPI template and resampling them to a resolution of 3 mm × 3 mm × 3 mm. (6) Smoothing: Applying a Gaussian filter with a full width at half maximum (FWHM) of 6 mm for spatial smoothing of the images.

#### 2.2.6 Resting-state activity analysis within ICNs

Group ICA (gICA) was performed using the GIFT software (SedDB, RRID: SCR_024416), a method for decomposing a set of images into statistically independent components. The process involved data decomposition, ICA computation, reconstruction of individual components, and Fisher z transformation. For each participant, The minimum description length (MDL) algorithm is used to determine the number of independent components (ICs) to 30, decompose 30 ics and generate independent spatial maps. IC selection was based on visual inspection and spatial correlation values between ICs and templates. Spatial maps of selected ICNs for each participant were converted to Z values, indicating their contribution to the temporal dynamics of the independent components. Resting-state brain network analyses for each group were performed using SPM 12 software in MATLAB. Firstly, the voxel single sample t test (P<0.05, FDR correction) was performed on the spatial maps of all subjects, and the independent component of the network were selected to obtain the corresponding mask. Statistical analysis (two-sample t test) was then performed for each component to compare the differences between the IGD and HC groups (P<0.05, FDR correction), using the mask obtained in the previous step.

#### 2.2.7 Functional connectivity analysis between ICNs

Multivariate analysis of covariance (Mancovan) module in GIFT software, Pearson correlation coefficients between brain functional networks in IGD group and HC group were calculated, respectively, which is functional network correlations (FNC), after Fisher Z transformation to ensure the normality of the data, the differences in FNC values between the groups were calculated (test level p < 0.05, FDR correction, two-tailed).

### 2.3 Statistical analysis

Statistical analysis was conducted using SPSS software (version 25.0, Chicago, Illinois) to compare the general and clinical characteristics of the two groups of participants, with a significance threshold set at P<0.05. Independent sample t-tests were used for continuous variables, and chi-square tests were utilized for categorical variables. Additionally, Pearson correlation analysis was performed to evaluate the association between scale scores and functional connectivity in both groups.

## 3 Results

### 3.1 Demographics and scales

The adolescent IGD group consisted of 44 participants, with 38 males and 6 females, while the HC group had 50 participants, including 40 males and 10 females. No significant differences were observed between the adolescent IGD and HC groups in terms of age, gender, and educational background. The scores on Young’s Internet Addiction Scale were significantly different between the two groups (P<0.05). On the Barratt Impulsiveness Scale, the adolescent IGD group exhibited significantly higher scores than the HC group in all three dimensions: motor impulsiveness, cognitive impulsiveness, and non-planning impulsiveness (P<0.05). The NEF and RSS scores also showed significant differences, with the adolescent IGD group scoring lower than the HC group in both measures (P<0.05). Additionally, there was a significant difference in CAMCS scores between the two groups, with the adolescent IGD group scoring higher than the HC group (P<0.05). Refer to **Table 1** for details.

**Table 1:** Demographic and clinical characteristics of participants.

	IGD(n=44)	HC(n=50)	T	P
Age (years)^a^	14.55 ± 2.02	14.70 ± 3.74	-0.253	0.801
Years of Education(years)^a^	8.75 ± 1.74	9.06 ± 2.37	-0.753	0.453
Gender(male: female)^b^	38∶6	40∶10	0.706	0.401
YIAS^a^	64.23 ± 10.82	25.58 ± 5.97	21.049	<0.001
BIS-11^a^	38.52 ± 3.27	22.02 ± 4.20	33.798	<0.001
Motor impulsiveness^a^	38.23 ± 3.53	22.30 ± 6.49	17.100	<0.001
Cognitive impulsiveness^a^	40.80 ± 5.80	21.99 ± 7.30	20.984	<0.001
Non-planning impulsiveness^a^	36.39 ± 8.00	21.60 ± 6.70	22.814	<0.001
NEF^a^	27.77 ± 0.94	30.76 ± 5.17	-13.405	<0.001
RSS^a^	6.61 ± 1.65	8.50 ± 0.99	-6.614	<0.001
D^a^	2.73 ± 1.34	0.54 ± 0.97	8.967	<0.001
CAMCS^a^	44.26 ± 5.44	32.11 ± 4.19	12.00	<0.001

^a^represents independent sample t-test, ^b^represents χ^2^ Inspection. YIAS, Young Internet Addiction Scale; BIS-11, Barratt Impulse Scale 11 Chinese Revised Edition; NEF, number of eye fixation; RSS, responsive of search scores; D, discriminant; CAMCS, the Chinese Adolescents’ Maladaptive Cognitions Scale.

### 3.2 Spatial distribution of ICNs

**Figure 1:**
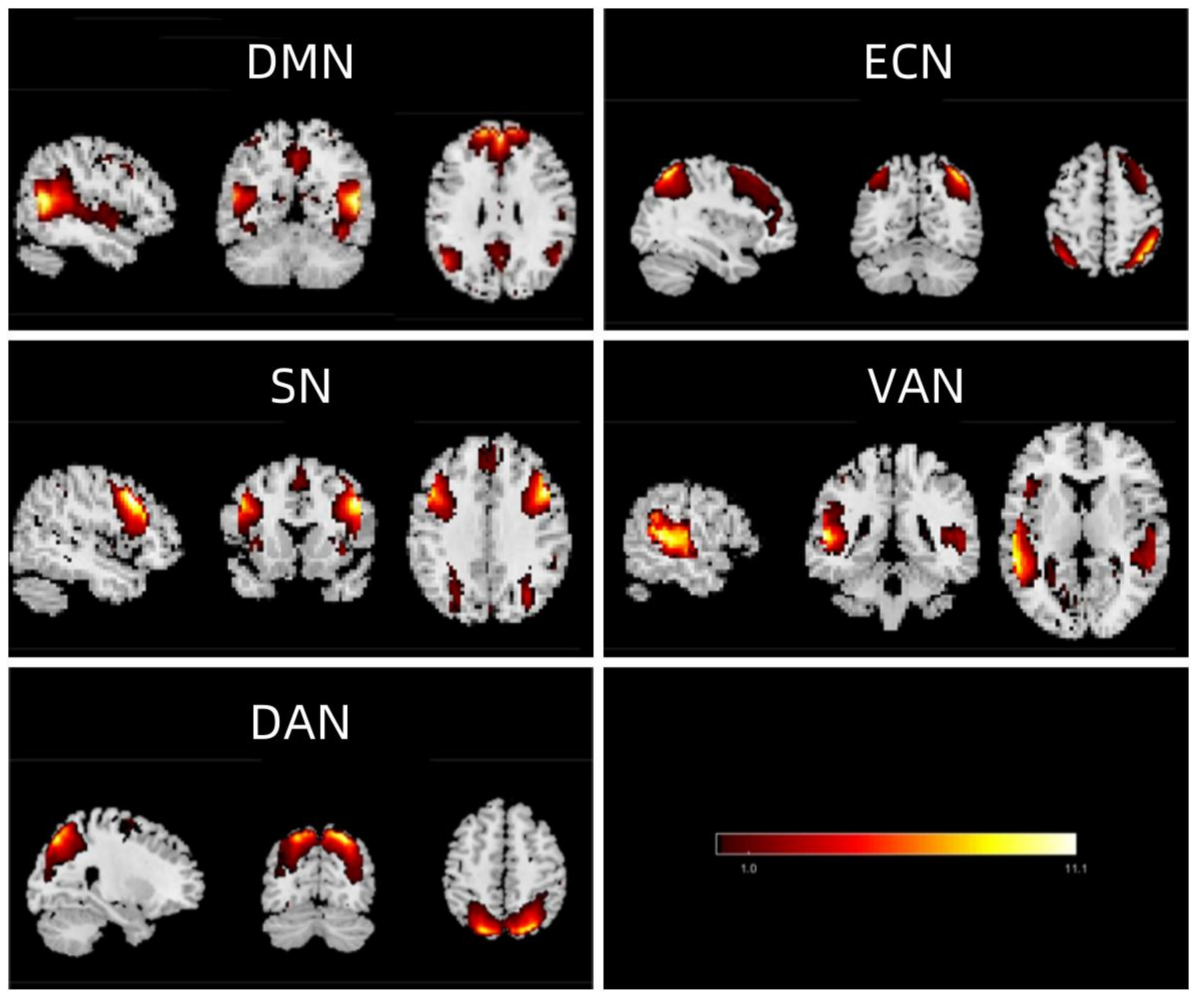
Extracted independent components. From the group ICA of resting-state data, five independent components were identified and classified as follows: DMN (default mode network), ECN (executive control network), SN (salience network), VAN (ventral attention network), and DAN (dorsal attention network).

From the 30 components derived using GIFT, five were selected based on our criteria: IC17, IC4, IC10, IC9, and IC15, which are represented in **Figure 1** as significant neural networks. The spatial maps of these five chosen ICN are depicted in **Figure 1**. Within these networks, the DMN consists of regions such as the medial prefrontal cortex, anterior cingulate cortex, posterior cingulate cortex, precuneus, and angular gyrus. The ECN encompasses areas like the medial prefrontal cortex, inferior frontal gyrus, and inferior parietal lobule, with the dorsolateral prefrontal cortex (dlPFC) being its central region. The SN comprises the insular cortex, dorsal anterior cingulate cortex, amygdala, and temporal pole. The VAN includes the ventral frontal cortex and temporo-parietal junction. Lastly, the DAN contains the bilateral intraparietal sulcus and the junction area of the precentral sulcus and superior frontal sulcus (frontal eye field).

### 3.3 Resting-state activity differences within ICNs related to adolescent IGD

**Figure 2:**
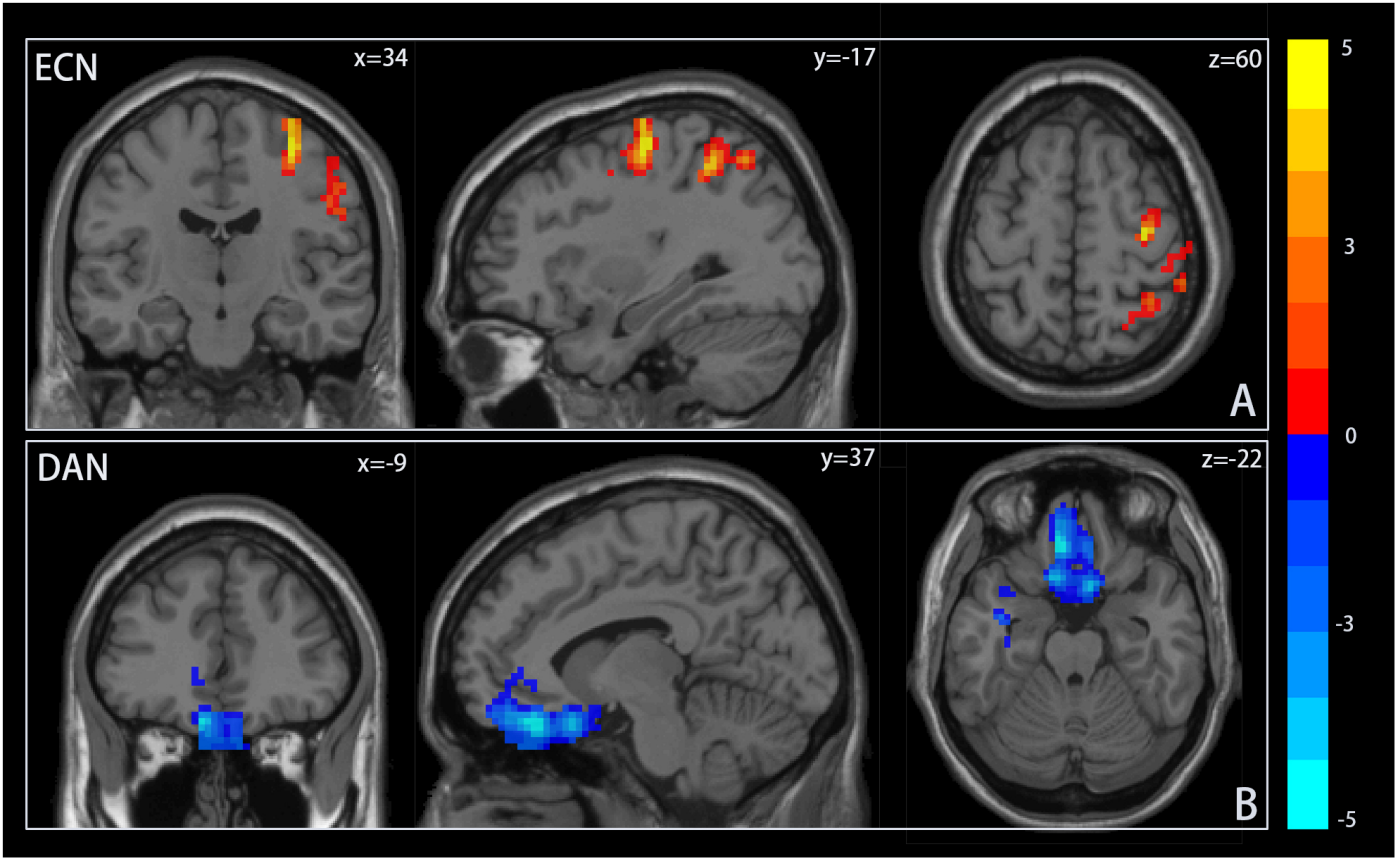
Resting-state activity differences within ICNs. Notes: **(A)** Resting state activity in the frontal gyrus and precentral gyrus within the executive control network(ECN) was significantly higher in the adolescent group with Internet Gaming Disorder (IGD) than in the healthy control group (HC). **(B)** In contrast, resting state activity in the frontal eye area within dorsal attention network(DAN) was significantly reduced compared to the HC group. Areas in red or blue indicate increased or decreased resting state activity, respectively.

To assess differences in resting-state activity within ICNs associated with adolescent IGD, a two-sample t-test was utilized. **Figure 2** displays brain regions with significant differences between groups. Adolescent IGD participants exhibited enhanced resting-state activity in the middle frontal gyrus (MFG) and precentral gyrus, part of the dLPFC, a key node of the ECN, compared to the HC group. In contrast, resting-state activity in the frontal eye field (FEF) of the DAN was diminished. Refer to **Table 2**. No differences in resting-state activity related to adolescent IGD were detected in other ICNs at the same significance threshold.

**Table 2:** Brain regions with differences in resting-state activity in the IGD group.

Abnormal brain area	ICN	Peak MNI coordinates	T	Voxel size
		X Y Z		
Middle frontal gyrus	ECN	44 33 42	3.2771	31
precentral gyrus	ECN	34 -17 60	4.3327	86
Frontal eye region	DAN	-9 37 -22	-3.6770	99

P<0.05, FDR correction, clump ≥ 30, MNI, Montreal Institute of Neurology; ECN, executive control network; DAN, dorsal attention network.

### 3.4 FC differences between networks related to adolescent IGD

**Figure 3:**
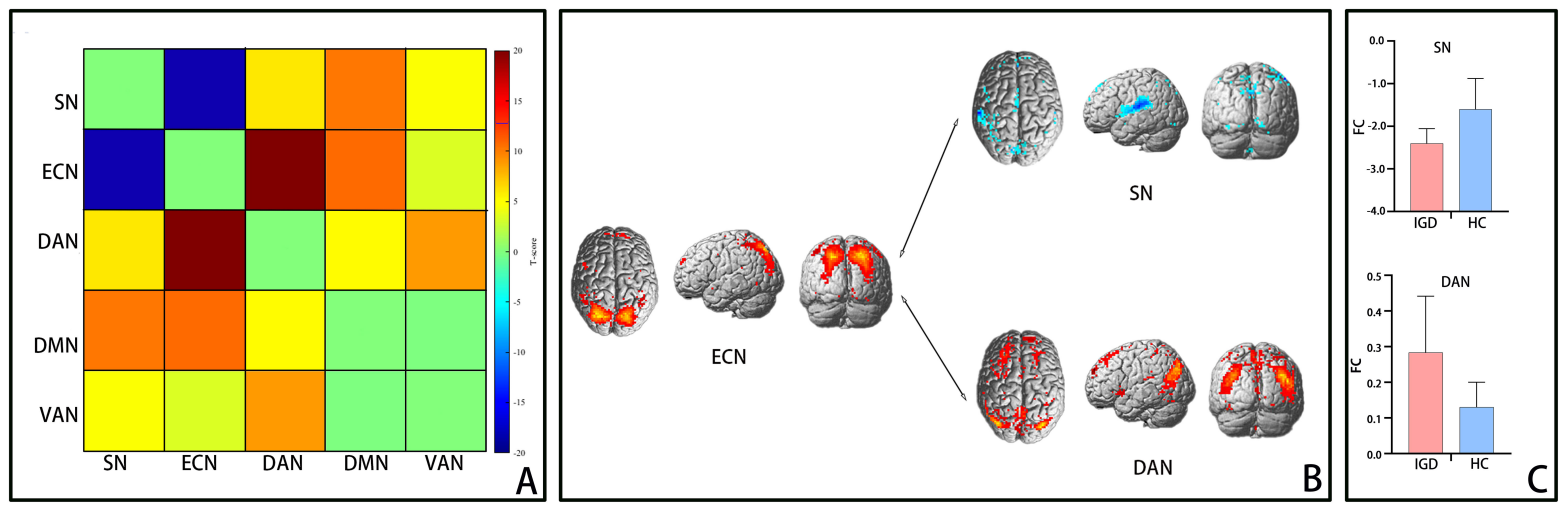
Functional connectivity differences between networks. Notes: **(A)** Heat map of functional connectivity between brain networks in the IGD group. Warm and cool colors represent areas with higher or lower functional connectivity between networks. **(B)** Display the statistical significance of functional connectivity between networks in brain 3D rendering of networks. The functional connectivity between ECN-SN decreased, while the functional connectivity between ECN-DAN increased (P<0.05, FDR corrected). **(C)** Use a two sample t-test to display the bar plot of functional connectivity between IGD and HC networks (P<0.05). The upper figure shows that compared with the HC group, the IGD group shows a decrease in the functional connections of ECN-SN, while the lower figure shows an increase in the functional connections of ECN-DAN. IGD, internet gaming disorder; HC, healthy controls; ECN, executive control network; SN, salience network; DAN, dorsal attention network.

**Figure 3** depicts the functional connectivity among all five networks. It was observed that in comparison to the HC group, the interaction between the ECN and SN was reduced(t = -3.1, p < 0.01), whereas the interaction between the ECN and DAN was heightened (t = 2.4, p < 0.01). No notable differences in interactions involving these networks were detected between the two groups (all p-values > 0.01), suggesting that these connections might not be linked to IGD.

### 3.5 Correlation analysis

**Figure 4:**
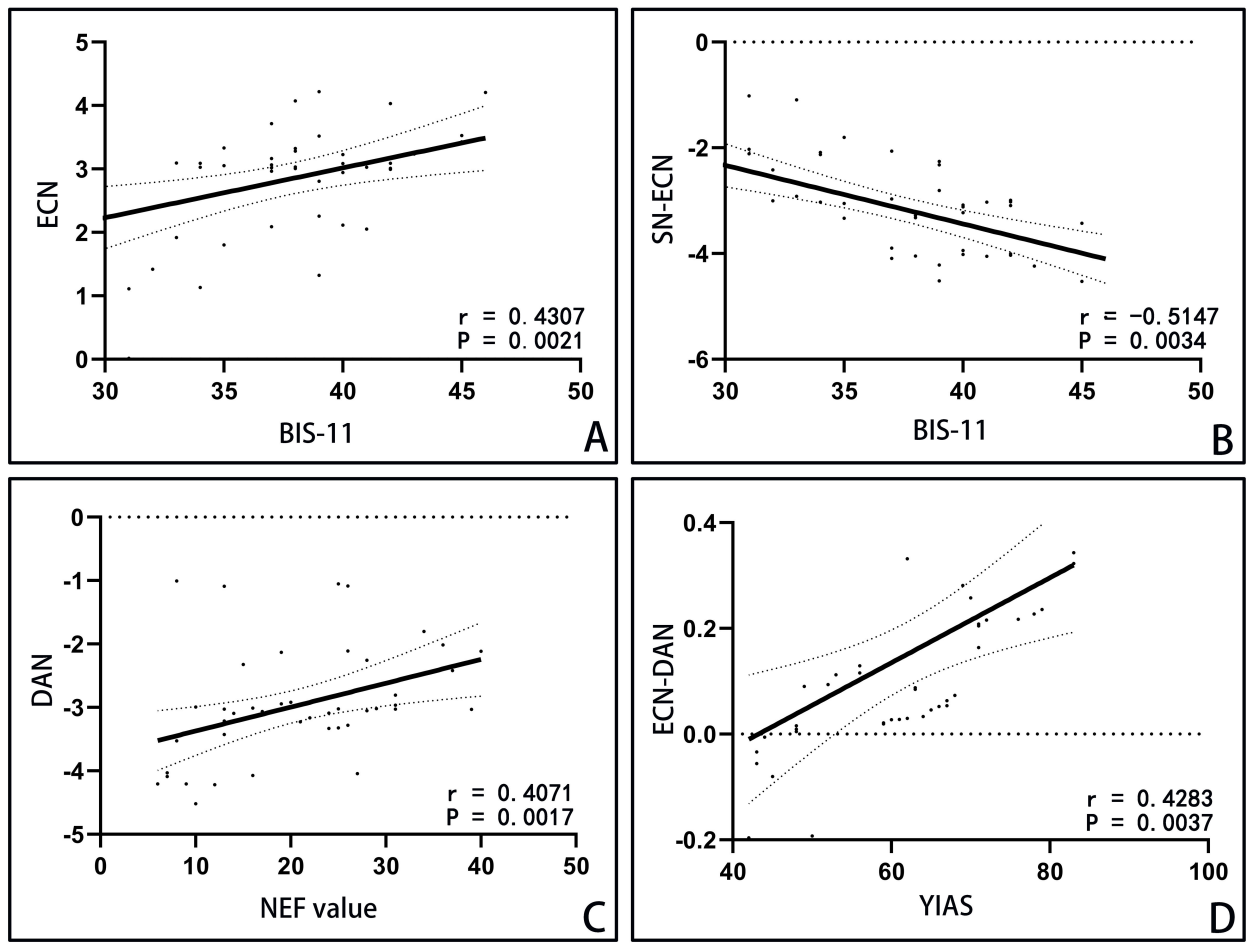
Correlation analysis in IGD group. Notes: **(A)** Abnormal resting-state activity of ECN was positively correlated with BIS-11 score. **(B)** Abnormal functional connections of SN-ECN were correlated with the score of BIS-11. **(C)** The abnormal resting-state activity of DAN was correlated with NEF value. **(D)** The abnormal functional connection strength of ECN-DAN was positively correlated with the total score of Young scale. ECN: executive control network; BIS-11: Barratt Impulse Scale 11 Chinese Revised Edition; SN: salience network; DAN, dorsal attention network; NEF, number of eye fixation.

Pearson correlation analysis (refer to **Figure 4**) in IGD group showed correlations between BIS-11 scores and three measures: YIAS scores(r=0.6350, P<0.0001), abnormal resting-state activity in ECN(r=0.4307,p=0.0021), and abnormal functional connectivity in SN-ECN(r=0.-5147,p=0.0034). The abnormal resting-state activity of DAN was correlated with NEF value(r=0.4071,p=0.0017), and the abnormal functional connectivity of ECN-DAN was correlated with YIAS score(r=0.4283, P=0.0037).

## 4 Discussion

Relative to the HC group, adolescents with IGD displayed increased resting-state activity within the ECN and reduced functional connectivity between the ECN and SN, linked to heightened impulsivity, which correlates positively with IGD severity. Studies have shown that participants with IGD exhibit enhanced functional connectivity in the ECN during cognitive tasks, which is related to a decline in the ability of IGD subjects to control impulsivity (27). Our results show that the CAMCS scores of the adolescent IGD group were higher than those of the HC group (P<0.05), indicating impaired cognitive function in adolescent IGD. In the Barratt Impulse Scale, the IGD group had the highest score for cognitive impulsivity factors and the highest standard deviation for unplanned impulsivity factors. This indicates that adolescents with IGD have higher variability in cognitive impulsivity and unplanned behavior, suggesting that the increased resting-state activity in the ECN cannot effectively suppress their impulsivity. One possible reason is that the ECN in adolescents is still not fully developed, and even if the resting-state activity within this network increases, it still cannot suppress impulsivity as effectively as in adults. Yuan et al. found that the interaction between the SN and the right ECN during the Stroop task was reduced in 28 IGD subjects compared to the control group (28). Our results show that the interconnection between the SN and ECN is reduced, indicating that adolescents with IGD cannot adequately suppress ECN activity in the switching process within the brain network during rest, leading to poor impulse control.

In comparison to the HC group, adolescents with IGD showed decreased resting-state activity in the frontal eye field (FEF) within the DAN and elevated functional connectivity between the ECN and DAN, linked to attentional deficits. The strengthened functional connectivity between the ECN and DAN was positively associated with the severity of IGD. The FEF is activated during finger pointing and saccade tasks (29). Eye movements are related to the control of attention and decision-making (30). Exploratory eye movement tests can serve as an objective behavioral indicator for detecting higher cognitive processes in the cerebral cortex and subcortex (31). NEF (number of eye fixations) and RSS (response exploration score) reflect an individual’s cognition, memory, and attention. Results showed that the NEF and RSS scores of the adolescent IGD group were significantly lower than those of the HC group (P<0.05), indicating attentional deficits related to weakened resting-state activity within the DAN. A possible explanation is that the extensive attentional shifts through multitasking, a characteristic of IGD, may impair cognitive function through habituation (32). The ECN is extensively connected with the DAN and plays a role in regulating perceptual attention (33). Studies have found (34) that reduced functional connectivity between the ECN and DAN is related to attentional development in adolescents with Attention-deficit/hyperactivity disorder (ADHD). Our study is the first to find increased functional connectivity between the ECN and DAN in adolescent IGD, related to attentional deficits. Dixon (35) et al.’s study found that the ECN can prioritize involvement in DAN connections during attentional deficits. Our results show that the DAN exhibits weakened resting-state activity, while the functional connectivity between the ECN and DAN is increased, related to attentional deficits in adolescent IGD subjects. Compared to adults, the effect size of attentional deficits in adolescents is lower (36). A possible reason is that the ECN in adolescents may not be as flexible as in adults in regulating prioritization of DAN connections, and attentional abilities continue to develop during adolescence. Fair et al. highlighted the significance of network segregation in the brain and cognitive development during adolescence (37), with attentional performance potentially improving as the ECN becomes more segregated from other brain networks. Enhancing the autonomy of the ECN and diminishing its connections with the DAN could contribute to the advancement of attentional development in adolescents. The functional connectivity between the ECN and DAN is positively correlated with the score on Young’s Internet Addiction Scale, suggesting that attentional deficits could result in increased functional connectivity between these networks, potentially leading to extended gaming behavior. This increased functional connectivity may serve as a predictor of the severity of adolescent IGD.

Brewer et al. found that decreased cognitive control ability can predict treatment outcomes and relapse of drug use (38). Therefore, understanding the neural mechanisms behind cognitive control in IGD is very important. Currently, there is limited understanding of the changes in connectivity within the ECN and between the ECN and DAN in behavioral and cognitive impairments (39). However, this seems to be a different area of functional connectivity in cognitive control for adolescent IGD. Changes in functional connectivity may specifically distinguish between adolescent IGD individuals with and without cognitive control impairments, and altered functional connectivity may predict the severity of adolescent IGD, which may be beneficial in developing specific treatment plans for adolescent IGD. Our study further demonstrates changes in the ECN, SN, and DAN and their interactions in individuals with adolescent IGD. Future treatments may enhance brain network connectivity through cognitive behavioral therapy (CBT) or external stimuli like transcranial magnetic stimulation, thereby enhancing the cognitive control ability of IGD (40), which may be an important goal of IGD treatment.

## 5 Limitations

This study has two limitations. Firstly, as a case-control study, it does not establish a causal link between adolescent IGD and compromised cognitive function. Longitudinal studies are required to explore this causal relationship. Secondly, the study predominantly involved male adolescents, potentially reflecting the higher incidence of IGD among males. Future research should examine the variations in resting-state brain networks of adolescent IGD across different genders.

## 6 Conclusion

This research demonstrates that changes in the ECN and DAN correlate with heightened impulsivity and attentional deficits in adolescents with IGD. The interaction between cognitive control disorders and resting-state brain networks in adolescent IGD is related.

## Data Availability

The raw data supporting the conclusions of this article will be made available by the authors, without undue reservation.
